# Adherence to Medication Among Diabetic Patients Attending the Non-communicable Disease Clinic of a Secondary Care Center in North Kerala, India

**DOI:** 10.7759/cureus.72408

**Published:** 2024-10-26

**Authors:** Mohamed Nizamudheen, Ameena Nisireen, Anupama Arumadi, Rupesh Raman, Jayakrishnan Thayyil

**Affiliations:** 1 Community Medicine, Kunhitharuvai Memorial Charitable Trust Medical College, Kozhikode, IND

**Keywords:** diabetes mellitus, diabetes treatment, kerala, management of diabetes, medication adherence

## Abstract

Introduction: People with diabetes mellitus can live longer and healthier lives if the disease is detected early and well-managed. The role of the doctor in educating the patient regarding diabetes management and that of the patient in taking responsibility for medication adherence and practicing proper self-care cannot be overemphasized. By examining the patterns and factors influencing adherence to medication, better insight can be gained into our understanding of controlling the disease. The present study aims to understand the adherence to medication among diabetic patients attending the non-communicable diseases (NCD) clinic of a secondary care center in Kerala, India.

Methods: This cross-sectional study was conducted among 100 diabetic patients attending the NCD clinic of a secondary care center in Kerala, India. Data was collected by interviewer-administered questionnaire and analyzed using Epi Info version 7.2 (Centers for Disease Control and Prevention, Atlanta, GA).

Results: Among the 100 study participants, 53% were females, and 84% had a family history of diabetes; 78% had at least one comorbidity. All participants had received advice regarding the importance of regular medication. Only 39% were regular in taking medication. Being male, unemployed, having a shorter duration of disease (less than one year), absence of comorbidities, shorter duration of drug use (less than two years), and taking a single medication were significantly associated with good medication adherence. In the logistic regression model, higher educational status, living without a partner, and longer medication duration indicated potential positive associations with adherence. Only 8% of participants had blood glucose levels under control.

Conclusions: This study highlights the challenges in diabetes management among patients in Kozhikode, Kerala. A significant proportion reported irregularities in their adherence to medication. The low percentage of participants with optimal blood glucose levels underscores the need for targeted interventions and personalized approaches to improve overall outcomes.

## Introduction

The prevalence of type 2 diabetes mellitus is increasing globally and has reached epidemic proportions in many countries. Around 422 million people worldwide have diabetes, the majority living in low and middle-income countries, and 1.5 million deaths are directly attributed to diabetes each year [1]. In India, an estimated 77 million people above the age of 18 years are suffering from type 2 diabetes mellitus and nearly 25 million are pre-diabetics. More than 50% of people are unaware of their diabetic status, which leads to health complications if not detected and treated early [2].

Kerala, a southern state in India, is unquestionably located in the highest epidemiologic transition zone, which has significantly impacted the state's morbidity and mortality figures. Regardless of the geography or economic strata, the state's rapid urbanization and industrialization have permeated down to its most basic levels, influencing people's lifestyles and creating an ideal environment for non-communicable diseases (NCD) to flourish [3].

People with diabetes mellitus can live longer and healthier lives if the disease is detected early and well-managed. Management using a standardized protocol can potentially prevent complications and premature death from diabetes using a small set of generic medicines, interventions to promote a healthy lifestyle, patient education to facilitate self-care, and timely treatment of complications. Facilities for diabetes diagnosis and management should be available in primary health care settings, with an established referral and back-referral system. In Kerala, under the National Program for NCD, a weekly/biweekly NCD clinic is functional from the primary level onwards. The present study aims to understand the adherence to medication among diabetic patients attending the NCD clinic of a secondary care center in Kerala, India. In a 2013 study in Kerala, 74% of patients were found to have poor medication adherence. Lower per capita monthly expenditure, irregular blood sugar monitoring, limited diabetes management instructions from health professionals, resorting to symptomatic management only, and those who did not receive family member's help to remember medications were more likely to report poor adherence compared with their counterparts [4]. More recent studies in the region are scarce and the present study aims to address this lacunae.

## Materials and methods

This cross-sectional study was conducted in the outpatient department of the NCD clinic of a secondary health care center in the Kozhikode district of Kerala, India. The study period was from 15th January to 1st March 2023.

Inclusion criteria

Patients diagnosed with type 2 diabetes mellitus for at least six months.

Exclusion criteria

Severely ill patients/patients with altered mental status/any patient unable to give valid consent.

Sample size

The sample size was calculated considering the adherence to anti-diabetic medication as 56.5%, with a relative precision of 20%, a confidence interval of 95%, and a non-response rate of 10%. After calculation, the minimum sample size was found to be 85 [5]. The sample obtained for the study was 100.

Data collection

Consecutive patients attending the clinic who fit the inclusion criteria were chosen for the study. The nature and objectives of the study were explained to the study participants and informed consent was taken. Data was collected by interview method using a pre-tested, semi-structured questionnaire, which was divided into the following sections: sociodemographic characteristics, general information about the disease, medications and adherence to it, and glycemic control (fasting blood sugar value: see Table 4 of Appendix).

Adherence to medication was determined by self-reporting by the participants, using an operational definition similar to another study conducted in India [6]. The definition of medication adherence missing one or fewer doses in the past two weeks was validated through cross-checking with participants' diaries, which documented their medication-taking behavior. All patients are instructed to maintain a medication diary upon diagnosis where they note down the date and time of taking medication; this is cross-checked by the healthcare provider on all review visits. This operational definition allowed for more consistent and reliable measurement of adherence, reducing potential recall bias associated with self-reporting.

All patients undergo a monthly fasting blood sugar (FBS) test at the hospital and this value is recorded in their OP diary. The latest FBS value was taken for the purpose of the study.

Statistical analysis

Data was cleaned, entered into an MS Excel sheet (Microsoft Corporation, Redmond, Washington, United States), and analyzed using Epi Info version 7.2 (Centers for Disease Control and Prevention, Atlanta, GA). Qualitative data are expressed in terms of frequencies and percentages, and quantitative data are expressed in terms of means, standard deviations, and median. The chi-square test (Pearson chi-square or Fischer's exact test) has been used to test the significance of relevant results involving qualitative data with a p-value of 0.05 being considered significant. A logistic regression model was used to check for potential predictors of adherence.

## Results

100 patients participated in the study. Their median age was 58 years with a range of 38-65 years. The sociodemographic and other baseline characteristics of the study population are shown in Table 1.

**Table 1:** Sociodemographic characteristics of the study participants

Sociodemographic characteristics	Variables	Frequency	Percentage
Age group	≤58 years	54	54
	>58 years	46	46
Gender	Male	47	47
	Female	53	53
Education	≤Elementary	24	24
	Middle school	39	39
	High school	34	34
	≥Graduate	3	3
Occupation	Unemployed	48	48
	Unskilled	3	3
	Semi-skilled	9	9
	Skilled	16	16
	Clerical/shop owner	10	10
	Semi-professional	4	4
	Professional	10	10
Relationship status	Living with partner	95	95
	No partner (unmarried/widowed/separated)	5	5
Duration of the disease	<1 year	24	24
	1-5 years	76	76
Family history	Nil	16	16
	First-degree relatives	74	74
	Second-degree relatives	10	10
Comorbidities	Hypertension	39	39
	Dyslipidemia	13	13
	Coronary artery disease	1	1
	Others	3	3
	>1 comorbidity	22	22
	Nil	22	22
Duration of taking medication	≤2 years	35	35
	>2 years	65	65
Types of drugs taken	Single oral hypoglycemic drug	26	26
	Insulin alone	1	1
	2 or more oral hypoglycemic drugs	58	58
	Insulin and oral hypoglycemic drugs	15	15

All the participants (100%) received advice regarding the importance of regular medication. They were asked to keep a diary for the same and record the date and time of taking the medication.

The median duration of taking medications was 36 months. 26 were on one oral hypoglycemic drug; 58 were on two or more oral hypoglycemic drugs. One was on insulin alone and 15 participants were taking insulin and an oral hypoglycemic drug. Only 39 participants were taking medication regularly, adhering to their doctor's advice. The common reasons for non-adherence to medication are shown in Table 2.

**Table 2:** Reasons for non-adherence to medication (n=61)

Reasons	Frequency	Percentage
Forgetfulness	34	55.7
Multiple drugs for different diseases	8	13.1
Hypoglycemic episodes	4	6.5
Drug unavailability	15	24.6

FBS level was satisfactory in only eight patients (i.e., <125mg/dL). 79 had FBS levels between 126 and 200 mg/dL; 13 had FBS above 200 mg/dL. The association of the various study variables to adherence to medication is given in Table 3.

**Table 3:** Association of various factors to adherence to medication ^*^p-value is significant. FBS: fast blood sugar

Variables	Good adherence (n=39)	Poor adherence (n=60)	𝛘² value	Odds ratio	95% confidence interval	P-value
Age						
≤58 years	21 (38.9%)	33 (61.1%)	0.012	0.95	0.42-2.14	0.9
>58 years	18 (40%)	27 (60%)				
Gender						
Male	23 (50%)	23 (50%)	4.04	2.31	1.02-5.27	0.04*
Female	16 (30.2%)	37 (69.8)				
Educational status						
Middle school and below	23 (37.1%)	39 (62.9%)	0.36	0.77	0.34-1.77	0.54
High school and above	16 (43.2%)	21 (56.8%)				
Occupational status						
Unemployed	35 (72.9%)	13 (27.1%)	5.03	2.59	1.12-6	0.02*
Employed	26 (51%)	25 (49%)				
Relationship status						
Living with partner	37 (39.4%)	57 (60.6%)	0.2	0.97	0.16-6.11	0.65
No partner	2 (40%)	3 (60%)				
Duration of the disease						
<1 year	17 (73.9%)	6 (26.1%)	24.68	6.95	2.42-19.96	0.0001*
≥1 year	22 (28.95%)	54 (71.05%)				
Family history of the disease						
Present	31 (37.35%)	52 (62.65%)	0.56	0.60	0.20-1.75	0.34
Absent	8 (50%)	8 (50%)				
Comorbidities						
Present	26 (33.3%)	52 (66.7%)	5.65	0.31	0.11-0.84	0.017*
Absent	13 (61.9%)	8 (38.1%)				
Number of drugs						
One OHA/Insulin alone	18 (69.23%)	8 (30.77%)	18.54	5.57	2.10-14.77	0.0002*
>1 drug	21 (28.77%)	52 (71.23%)				
Duration of taking medication						
≤2 years	23 (67.65%)	11 (32.35%)	21.79	6.40	2.57-15.97	0.00003*
>2 years	16 (24.62%)	49 (75.38%)				
FBS levels						
<125 mg/dL	5 (12.8%)	3 (4.9%)	2.84	1.06	0.64-12.65	0.256
>125 mg/dL	34 (87.2%)	58 (95.1%)				

A significant association was seen with gender (males>females), employment status (unemployed>employed), duration of the disease (duration of disease less than one year>duration more than one year), comorbidity status (no comorbidities>comorbidities present), number of drugs (single drug>multiple drugs), duration of taking medication (duration less than two years> duration more than two years), and medication adherence.

A logistic regression analysis was conducted to examine factors associated with regular medication adherence. The model included age group, gender, educational status, occupational status, relationship status, duration of illness, family history, comorbidities, medication duration, and number of medications as independent variables. While none of the variables reached statistical significance at the p<0.05 level, several factors showed potentially meaningful associations. Higher educational status (OR=2.15, 95% CI: 0.68-6.75), living without a partner (OR=3.18, 95% CI: 0.64-15.85), and longer medication duration (OR=3.18, 95% CI: 0.88-11.46) were associated with increased odds of regular medication adherence, albeit not statistically significant.

## Discussion

Kerala has become the hub of NCD due to various factors like rampant modernization and urbanization, high consumerism, and fund flow from abroad [7]. As diseases require life-long management, adherence to treatment and self-care practices are important in patients with NCD to ensure the prevention of life-threatening complications and maintain quality of life. The present cross-sectional study was conducted in a secondary care center in Kerala, India to understand the adherence to medication among diabetic patients. 100 patients attending the NCD clinic of the hospital were included in the study.

In the present study, only 39.4% were taking regular medication, which is an alarming finding. Forgetfulness (54%) and unavailability of the drugs (24.6%) at the pharmacy of the hospital were cited as the main reasons for irregular medication. Being male, being unemployed, duration of disease less than one year, absence of comorbidities, and taking a single drug were found to be significantly associated with good medication adherence.

In similar studies conducted in South India, medication adherence was found to be slightly better (56.5% and 49.3%); a study in Andhra Pradesh even reported an adherence of 90.3% [6,8,9]. Good medication adherence in the study conducted in Mangaluru was found to be associated with age (<60 years), female gender, and being married, while in a Puducherry study, it was associated with increasing age(>60 years) and longer duration of the disease [8,9]. In a 2019 study conducted in Basra, medication adherence was found to be 65.4%, while a study in Erbil, Iraq, using the Morisky Medication Adherence Scale-8, reported that 40.3% of participants had poor adherence [10,11]. In the Erbil study, poor adherence was significantly associated with lower education levels and unemployment, highlighting the impact of sociodemographic factors on medication-taking behavior. The differences among these studies emphasize the importance of using less complicated regimens, using reminder systems (like setting an alarm on the phone), and continued support from the health system to improve adherence.

In the present study, adequate glycemic control was seen only in 8%, whereas in another study conducted in Ethiopia, it was 41.8%. The main external factor for non-adherence in this study was lack of finance (37.1%), whereas in the present study, it was forgetfulness (55.7%) [12]. In the Andhra Pradesh study, poor glycemic control was seen in 42%, and the asymptomatic nature of the disease and the high cost of treatment were the main barriers to adherence [6]. Despite Kerala's greater patient education and healthcare availability, our study's glycemic control rate was lower than similar studies, indicating that adherence hurdles may differ greatly across different settings. While financial constraints were a major factor in these studies, forgetfulness being the primary reason in our study indicates that non-adherence in Kerala may be more behavioral, highlighting the need for strategies such as patient reminders and adherence counseling.

The study identified significant associations between medication adherence and sociodemographic factors such as gender, employment status, disease duration, and comorbidity status. Although logistic regression did not yield statistically significant predictors, higher educational status, living without a partner, and longer medication duration indicated potential positive associations with adherence. The study highlights significant gaps in adherence to medication among diabetic patients in Kerala. Addressing these issues requires targeted interventions such as simplifying treatment regimens, improving drug availability, and enhancing support systems. By focusing on these areas, healthcare providers can better manage NCD and improve the quality of life for patients.

Limitations

The study was reliant on self-reported adherence, which may potentially introduce recall bias. Diary entries offered a cross-check, but they could still contain errors if individuals forgot to enter dosages or change entries to match how adherence is viewed, potentially leading to an overestimation of adherence rates. Our study was done in a single center and this could affect the generalizability of the findings.

## Conclusions

The study reveals critical gaps in the management of diabetes in the state of Kerala in India. Adherence to medication was found to be low. Adequate glycemic control was seen in only 8%. Being male, being unemployed, having a lesser duration of disease (<1 year), having an absence of comorbidities, having a lesser duration of taking drugs (<2 years), and taking a single drug were found to be significantly associated with good medication adherence. Poor compliance with medication was mainly due to the patient's forgetfulness and lack of drug availability; this highlights the need for improved patient education and support systems. Creating a support group for the patients under the guidance of the healthcare provider or a local influencer may help in improving adherence.

As a chronic disease requiring lifelong management and the potential to cause life-threatening complications, continuous patient motivation and involvement of support systems (families, friends, and support groups) as well as enhancing the capacity of health care systems are necessary to achieve adequate glycemic control and ensure the quality of life of diabetic patients.

## Appendices

**Table 4:** Adherence to medication among diabetic patients attending the NCD clinic of a secondary care center in North Kerala, India NCD: non-communicable disease

Section	Question	Options/space for answer
A: Sociodemographic Characteristics		
1	Name:	________________________________
2	Age:	________ years
3	Gender:	Male/Female/Other
4	Educational Status:	Graduate and above/High school (9-12th)/Middle school (5-8th)/<5th standard No formal education
5	Occupation:	__________________________
6	Relationship Status:	Living with partner/No partner (unmarried/widowed/separated)
B: General Information About the Disease		
7	Duration of Disease:	<1 year /1-5 years
8	Family History of Diabetes:	Nil/First-degree relatives (parents/siblings)/Second-degree relatives (grandparents/uncles/aunts)
9	Comorbidities (Select all that apply):	Nil/Hypertension/Dyslipidemia/Coronary artery disease/Others (please specify): _________________
C: Medications and Adherence		
10	Duration of Taking Medication:	__________ (in months)
11	Types of Drugs Taken:	Single oral hypoglycemic drug /Insulin alone/2 or more oral hypoglycemic drugs /Insulin and oral hypoglycemic drug(s)
12	Doctor advised Regular Medication:	Yes / No
13	Adherence to Regular Medication (defined as missing one or fewer doses in the past two weeks):	Yes / No
14	If not adherent, reasons for non-adherence (Select all that apply):	Forgetfulness/Multiple drugs for different diseases/Hypoglycemic episodes/Drug unavailability/Other (please specify): ________
D: Glycemic Control		
15	Latest Fasting Blood Sugar Value:	<125 mg/dL/126-200 mg/dL />200 mg/dL
E: Additional Information		
16	Additional comments or information:	
